# Determining Herd Immunity Thresholds for Hepatitis A Virus Transmission to Inform Vaccination Strategies Among People Who Inject Drugs in 16 US States

**DOI:** 10.1093/cid/ciad552

**Published:** 2023-09-21

**Authors:** Judy Yang, Nathan C Lo, Emmanuelle A Dankwa, Christl A Donnelly, Ribhav Gupta, Martha P Montgomery, Mark K Weng, Natasha K Martin

**Affiliations:** Division of Infectious Diseases and Global Public Health, University of California–San Diego, La Jolla, California, USA; Division of Infectious Diseases and Geographic Medicine, Department of Medicine, Stanford University, Stanford, California, USA; Center for Communicable Disease Dynamics, Department of Epidemiology, Harvard T. H. Chan School of Public Health, Boston, Massachusetts, USA; Department of Statistics, University of Oxford, Oxford, United Kingdom; Medical Research Council Centre for Global Infectious Disease Analysis, Imperial College London, London, United Kingdom; Division of Infectious Diseases and Geographic Medicine, Department of Medicine, Stanford University, Stanford, California, USA; University of Minnesota Medical School, Minneapolis, Minnesota, USA; Division of Viral Hepatitis, US Centers for Disease Control and Prevention, Atlanta, Georgia, USA; Division of Viral Hepatitis, US Centers for Disease Control and Prevention, Atlanta, Georgia, USA; Division of Infectious Diseases and Global Public Health, University of California–San Diego, La Jolla, California, USA; Population Health Sciences, University of Bristol, Bristol, United Kingdom

**Keywords:** hepatitis A, critical vaccination coverage, people who inject drugs, herd immunity, dynamic modeling

## Abstract

**Background:**

Widespread outbreaks of person-to-person transmitted hepatitis A virus (HAV), particularly among people who inject drugs (PWID), continue across the United States and globally. However, the herd immunity threshold and vaccination coverage required to prevent outbreaks are unknown. We used surveillance data and dynamic modeling to estimate herd immunity thresholds among PWID in 16 US states.

**Methods:**

We used a previously published dynamic model of HAV transmission calibrated to surveillance data from outbreaks involving PWID in 16 states. Using state-level calibrated models, we estimated the basic reproduction number (*R*_0_) and herd immunity threshold for PWID in each state. We performed a meta-analysis of herd immunity thresholds to determine the critical vaccination coverage required to prevent most HAV outbreaks among PWID.

**Results:**

Estimates of *R*_0_ for HAV infection ranged from 2.2 (95% confidence interval [CI], 1.9–2.5) for North Carolina to 5.0 (95% CI, 4.5–5.6) for West Virginia. Corresponding herd immunity thresholds ranged from 55% (95% CI, 47%–61%) for North Carolina to 80% (95% CI, 78%–82%) for West Virginia. Based on the meta-analysis, we estimated a pooled herd immunity threshold of 64% (95% CI, 61%–68%; 90% prediction interval, 52%–76%) among PWID. Using the prediction interval upper bound (76%) and assuming 95% vaccine efficacy, we estimated that vaccination coverage of 80% could prevent most HAV outbreaks.

**Conclusions:**

Hepatitis A vaccination programs in the United States may need to achieve vaccination coverage of at least 80% among PWID in order to prevent most HAV outbreaks among this population.

## Teaser

Hepatitis A vaccination programs in the United States will need to achieve vaccination coverage of at least 80% among people who inject drugs in order to prevent most HAV outbreaks among this population.

Hepatitis A virus (HAV) outbreaks associated with person-to-person transmission continue across the United States and globally [1–7]. Since 2016, in the United States, more than 44 900 outbreak-associated cases across 37 states have occurred, resulting in more than 420 deaths as of August 2023 [1]. The primary identified risk group for HAV infection is people who use drugs (PWUD) [1]. HAV is transmitted through the fecal–oral route, through close contact with an infected person, or through contaminated food or water. Infection with HAV causes acute hepatitis, typically characterized by a clinical presentation of fatigue, nausea, emesis, abdominal pain, diarrhea, anorexia, jaundice, and fever; in some cases, it can lead to hospitalization, liver failure, and death [4, 8, 9].

Hepatitis A vaccines are highly efficacious, offering 90%–95% protection with 2 doses [10, 11]. In 1996, hepatitis A vaccination was introduced in the childhood immunization schedule in the United States for children aged ≥24 months in high-burden communities and for adults with increased risk for HAV infection or severe disease from HAV. In 2006, the Advisory Committee for Immunization Practices’ (ACIP) recommendations expanded to include vaccination of all children aged 12–23 months, regardless of risk category or location [12]. Therefore, although vaccination coverage among adolescents (aged 13–17 years in 2021) is 85% (2-dose coverage [13]), it is substantially lower among adults (aged ≥19 years in 2018; 2-dose coverage, 11.9% [14]). Additionally, due to recognition of PWUD (injection and noninjection) as a population at high risk of HAV infection, PWUD have been recommended for vaccination since 1996 [12], yet vaccination coverage among this group remains suboptimal [15–17]. Transmission between PWUD can occur through multiple routes, such as close contact with lack of access to hygiene facilities, housing, and barriers to obtaining clean supplies, in addition to potential modes of ingestion of contaminated drugs, sharing contaminated supplies, and sexual contact [18]. Among this population, recent estimates indicate that more than 50% of people who inject drugs (PWID) remain at risk for HAV infection [15–17]. Given the high and ongoing number of HAV outbreaks among PWID (a subset of PWUD) in the United States, this population remains of particular public health concern regarding provision of appropriate prevention efforts.

Despite efforts to increase vaccination among PWID in settings with ongoing HAV outbreaks, little is known about the critical vaccination coverage required to prevent future outbreaks in this population. One previous study [19] in Louisville, Kentucky, found a basic reproduction number (*R*_0_) of between 2.85 and 3.54 for the 2017–2019 outbreak, corresponding to an estimate of 76% (95% confidence interval [CI], 72%–80%) for the critical vaccination coverage level required to prevent further outbreaks among PWUDs or who experienced homelessness (assuming a 90% vaccine efficacy). However, this estimate was in a single geographic setting, and there was wide uncertainty.

To address this gap, we used surveillance data from hepatitis A cases with injection drug use as a reported risk factor from 16 states that experienced hepatitis A outbreaks associated with person-to-person transmission combined with epidemic modeling to estimate the herd immunity thresholds among PWID in these states to inform future vaccine policy and implementation.

## METHODS

### Overview

We used a previously published dynamic model of HAV transmission with a single risk group [19]. The model was calibrated separately to surveillance data from hepatitis A cases with injection drug use as a reported risk factor from 16 states that experienced hepatitis A outbreaks associated with person-to-person transmission. Using these 16 separately calibrated models, we estimated the *R*_0_ and herd immunity thresholds for PWID in each state. We then performed a meta-analysis of herd immunity thresholds to determine the critical vaccination coverage required to prevent HAV outbreaks among PWID in 16 US states.

### Surveillance Data

We used surveillance data from the Centers for Disease Control and Prevention National Notifiable Diseases Surveillance System (NNDSS) from 2016 through 2019. HAV cases were categorized based on the 2012 and 2019 US Council of State and Territorial Epidemiologists case definitions [20, 21]. For each reported case, data were collected on reporting date, county of residence, age, sex, injection drug–use status (yes or no), and clinical outcome (hospitalization, death). We selected states from the NNDSS data with sufficiently large outbreaks that involved PWID (first, we identified the peak month of cases where injection drug use status was “yes” and selected states where the peak month of PWID cases was >10), yielding 16 states for inclusion: Alabama, Arkansas, Florida, Indiana, Kentucky, Louisiana, Massachusetts, Mississippi, New Mexico, New York (excluding New York City), North Carolina, Ohio, Tennessee, Utah, Virginia, and West Virginia. Other states with no risk factor data or small outbreaks (≤10 PWID cases in peak month) involving PWID were excluded. For our primary analysis in each state, we used data from cases with injection drug use status reported as “yes,” and we explored the impact of missing injection drug use risk factor data in our sensitivity analysis.

### Model

We used a previously published compartmental dynamic transmission model of HAV [19] to simulate transmission among PWID. The PWID population was disaggregated into 5 mutually exclusive compartments: susceptible (S), latent (L), infectious (I), temporary remission (R), or immune (Z). Model equations are in the Supplementary Material and described briefly as follows. At the onset of the epidemic, a proportion of individuals are immune to disease due to vaccination and/or past infection. At a given rate, susceptible individuals become infected with HAV, moving to the latent stage where they are infected but not infectious. Individuals then transition to the infectious stage. We assume homogenous mixing of infectious persons with susceptible persons for the contact structure within the population, with model calibration at the state level. Subsequently, infectious persons either recover permanently or enter the temporary remission stage. Individuals who recover permanently move to the immune stage. Temporary remission followed by relapse occurs in about 10%–15% of cases, lasting 4–8 weeks until they relapse and return to the infectious stage [22, 23]. The model does not account for hepatitis A–related deaths due to the low hepatitis A mortality rate observed among the risk group (approximately 1 death per 100 cases [4]). The model does not include background mortality due to the relatively short duration simulated during the initial outbreak stage (on the order of months).

### Model Parameterization and Calibration

Model parameters and their uncertainty are described in Supplementary Tables 1 and 2. Natural history parameters for HAV infection were obtained from published studies. The population of PWID was estimated from a national population size estimate of 3.7 million PWID age 18 + or above in 2018 [24]. Because state-level PWID population size estimates are not readily available, we made the simplifying assumption that the distribution of PWID by state is similar to the distribution of people who use illicit drugs excluding marijuana by state. We therefore allocated the 3.7 million PWID by state using a weighting based on the state-level distribution of the number of people age 18 + with illicit drug use excluding marijuana from the Substance Abuse and Mental Health Services Administration National Survey on Drug Use and Health 2017/2018 [25]. The number of people with illicit drug use by state was obtained by multiplying the state-level prevalence of illicit drug use in the past month excluding marijuana for age 18 + by the state population size age 18 + in 2018 [26], giving the total number of PWUD in each state, which was then used to calculate the proportion of PWUD by state. We then multiplied the national PWID population size estimate by the state-level proportion of PWUD to obtain the state-level PWID population estimate. Estimates for the proportion of PWID immune to hepatitis A at the start of the epidemic were informed by recent estimates among PWID in Wisconsin and Philadelphia [15, 16].

The model was calibrated for the initial outbreak stage until the epidemic peak in each state using monthly case count data of detected cases among PWID. Assuming observed case counts were Poisson-distributed, model calibration was performed using maximum likelihood estimation, generating estimates of the effective contact rate at the start of the epidemic and the initial number of infected individuals. Simultaneous 95% profile likelihood–based CIs were obtained for all estimated parameters, using Latin hypercube sampling to incorporate uncertainty in the PWID population size (N). Uncertainty estimates for the infection trajectory were calculated using Latin hypercube approximation of the parameter space confidence region.

### Reproduction Number and Herd Immunity Threshold

We calculated the basic reproduction number, *R*_0_, defined as the expected number of secondary infections caused by a single infected individual in an entirely susceptible population. For this estimate, we used the calibrated force of infection term (β), recovery rate (γ), and proportion of recovery (*η*). Using the next-generation matrix method,


$$R_{0}=\;\frac{\beta }{\eta \gamma }.$$

The herd immunity threshold (*ψ*) is given by *ψ* = 1 − 1*/R*_0_, assuming homogenous mixing. When the vaccine efficacy is less than 100%, the critical vaccination coverage level (*V_c_*) is *V_c_* = ψ/ω*, where ω* represents the full series vaccine efficacy among PWID. We set ω* at 95% based on 2-dose vaccine efficacy estimates in this range [9].

As the estimation of *R*_0_ and *ψ* is based on the initial outbreak period of the epidemic, we focus on modeling this period only and neglect the impact of mass vaccination efforts that occurred after the onset of the epidemics.

### Meta-analysis

We performed a meta-analysis of the herd immunity threshold in Stata [27] using metaprop, a package to perform meta-analysis of proportions, assuming random effects. We present the pooled estimate of herd immunity threshold and I^2^ statistic (as a measure of heterogeneity, noting it should be interpreted with caution for meta-analyses of prevalence, where heterogeneity is generally high [28]). The pooled estimate presents the average herd immunity across the states, but we additionally calculated the prediction interval [28–30] to give a range for the predicted herd immunity threshold if there were another HAV outbreak among PWID in 1 of the states simulated (or a setting similar to those simulated). In determining a threshold vaccination target, we calculate the 90% prediction interval (interval from 5%–95%); we chose the 90% interval as there would be a 95% chance the herd immunity threshold would fall under the upper limit of the 90% prediction interval based on the analysis.

### Sensitivity Analyses

We performed sensitivity analyses to evaluate the robustness of the study findings. To address missing data on PWID status, we repeated the analysis including a proportion of the HAV cases with an unknown PWID status based on the overall proportion of cases among PWID versus non-PWID in each state. For example, if *P*% of all cases in a state (during 2016–2019) with drug use risk factor data reported were among PWID, we reclassified *P*% of the monthly cases with unknown injection drug use risk as PWID for the sensitivity analysis. For each state, after the unknown cases were reallocated, the models were recalibrated and the impact on *R*_0_ and critical vaccination coverages was assessed. Despite this missingness, we note that for all 16 states, there was evidence of routine collection of injection drug use risk data prior to the outbreak start. We additionally performed alternative specifications to the meta-analysis, including performing the analysis on the *R*_0_ estimates instead of herd immunity thresholds.

This study was approved by the University of California–San Diego Institutional Review Board and deemed nonhuman subjects research given the use of deidentified data.

## RESULTS

**Figure 1.**
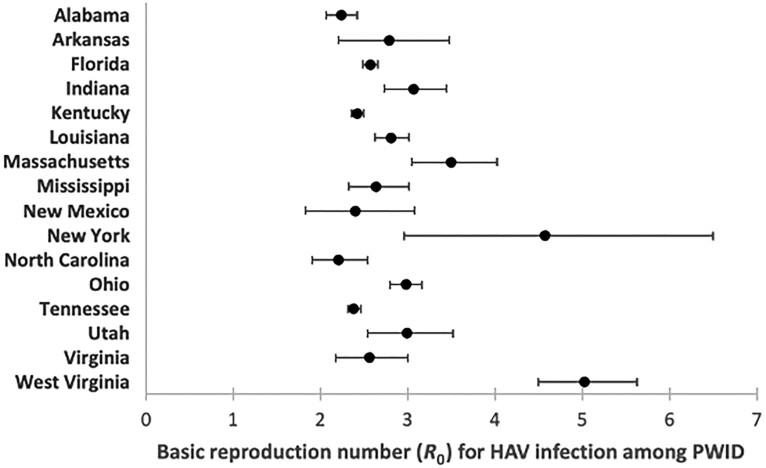
Estimated basic reproduction number (*R*_0_) for HAV infection among PWID in 16 US states. Dots represent median estimates, with whiskers indicating 95% confidence intervals. Abbreviations: HAV, hepatitis A virus; PWID, people who inject drugs.

In the 16 states during 2016–2019, there were 9085 cases among PWID for inclusion in the analysis. The calibrated models fit qualitatively well to the surveillance data for each of the 16 states (Supplementary Figure 1). Estimates of *R*_0_ across states with outbreaks between 2016 and 2019 ranged from a median estimate of 2.2 for North Carolina (95% CI, 1.9–2.5) to as high as 5.0 (95% CI, 4.5–5.6) for West Virginia. However, most median *R*_0_ estimates (13 of 16) fell between 2.2 and 3.1 (Figure 1), with West Virginia and New York having higher estimates (median above 4.0) and New York having wide uncertainty bounds.

**Figure 2.**
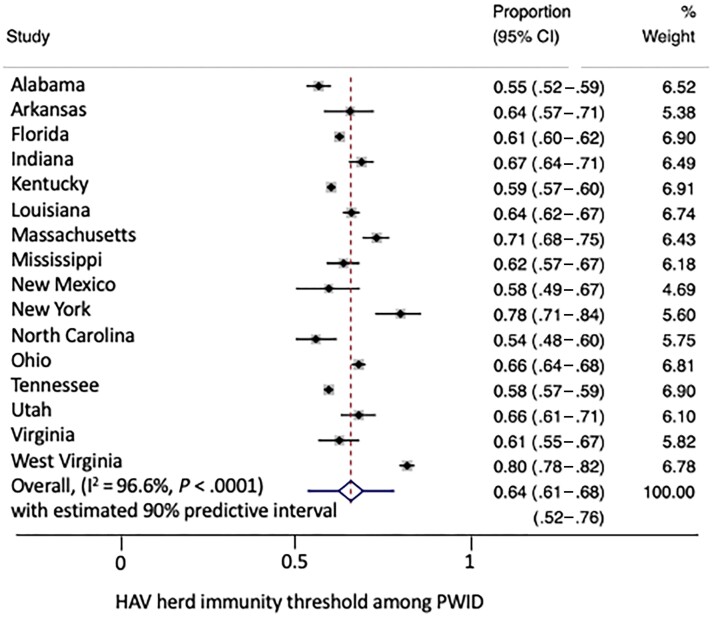
Meta-analysis of herd immunity thresholds for HAV infection among PWID. % weight denotes the weighting of the study in the meta-analysis. Abbreviations: CI, confidence interval; HAV, hepatitis A virus; I^2^, I^2^ statistic variance estimator; PWID, people who inject drugs.

The corresponding herd immunity thresholds (Figure 2) ranged from as low as 55% (95% CI, 47%–61%) for North Carolina to as high as 80% (78%–82%) for West Virginia. Most median herd immunity thresholds ranged from 55% to 67% (for 13 of 16 states).

The meta-analysis estimated an average overall herd immunity threshold of 64% (95% CI, 61%–68%, I^2^ 96%). The 90% prediction interval was 52%–76%, indicating that if there was another hepatitis A outbreak in 1 of these states or a “similar” state, there would be a 95% chance the herd immunity threshold would fall below 76%. This corresponds to a target critical vaccination threshold of 80% among PWID in these 16 states, assuming 95% vaccine efficacy.

As a substantial number of additional cases in these states had unknown or missing data for drug use (n = 6337, 40% of all cases), we performed a sensitivity analysis to reclassify a proportion of cases with unknown risk factor data as PWID. This resulted in negligible change in the estimated herd immunity thresholds for each state, with <1% difference in all states except New Mexico (−2%) and New York (−4%; Supplementary Figure 2). An alternative approach to performing the meta-analysis on the *R*_0_ estimates (Supplementary Table 3) and then calculating the herd immunity threshold based on the pooled *R*_0_ generated an average herd immunity threshold of 65%.

## DISCUSSION

In this study, we found that hepatitis A vaccination coverage of more than 80% may be required to reliably prevent most HAV outbreaks among PWID in the 16 US states examined. Our findings suggest that a relatively high population immunity level is required to prevent HAV outbreaks among PWID, suggesting the need for enhanced implementation of vaccination strategies in this population. The population of PWID remains at high risk for many infectious diseases, including HAV. While ACIP guidelines have included injection and noninjection drug use as an indication for vaccination since 1996 [12], the population of PWID remains undervaccinated. A 2018 study among PWID with hepatitis C in Wisconsin found that 58% had not received any dose of hepatitis A vaccine [16], and another study in the same year among PWID in Philadelphia found that 49% were susceptible to HAV infection based on serological testing [15]. In the National Health and Nutrition Examination Survey, among PWID during 2011–2016, 71% were susceptible to HAV infection, with no significant change over time [17].

Therefore, our study supports the need for intensive efforts to increase vaccination coverage in PWID whether in the clinical setting through primary care or emergency department visits or efforts for active vaccination programs with community partners, such as syringe service programs [31–33]. Despite ongoing outbreaks, vaccination programs have been hampered by vaccine cost, insufficient workforce, and high stigmatization of communities such as PWID [34]. Successful models included those that built strong multisectoral partnerships, such as among social service organizations, law enforcement agencies, correctional facilities, and emergency departments. Enhanced approaches such as targeted outreach, social media, and word-of-mouth communication through trusted community leaders are required to establish trust and community engagement [34]. Over time, vaccination coverage among PWID is likely to increase as the younger population receives routine HAV vaccination in childhood under current recommendations into adulthood.

Our study findings are consistent with those from prior work. A previous study of critical HAV vaccination coverage among PWUD and/or people experiencing homelessness in Louisville [19] estimated a critical vaccination threshold of 76% (95% CI, 72%–80%) assuming a 90% vaccination efficacy, which is similar to our findings. However, our study has the strength of including data among PWID from 16 states across diverse settings. Of note, while we found that vaccination coverage of at least 80% among PWID was needed in order to prevent most HAV outbreaks, this estimate was based on the upper bound of the prediction interval, and there were states with higher values (eg, West Virginia) that may require higher vaccination coverage.

Our study has limitations. First, our study is limited to PWID. People who use noninjection drugs are at risk for HAV infection. However, due to a lack of reporting of noninjection drug use in our dataset, we were unable to examine this factor. Nevertheless, our results are similar to those from our previous analysis among PWUD and persons experiencing homelessness in Kentucky, indicating that similarly high levels of vaccination coverage are required among both injection and noninjection PWUD. Additionally, we simulated a single risk population of PWID and did not explicitly account for other potential risks or interactions with other risk groups. Indeed, only a very small number of cases (64 of 9065, 0.7%) reported both PWID and male-to-male sexual contact as risk factors. However, it is possible that male-to-male sexual contact as a risk factor was underreported, resulting in misclassification of risk. Given the outbreaks among men who have sex with men in the United States, additional analyses that focus on critical vaccination coverage in this group are warranted. Second, there was an important number of cases with unknown or missing risk factor information related to injection drug use, although our sensitivity analyses indicated that our study findings on herd immunity thresholds were robust to this missing data. We additionally confirmed that risk factor information was routinely collected prior to the observed outbreaks, so we did not see conclusive evidence for changing risk ascertainment over time. Nevertheless, changes in case ascertainment and/or risk factor over time during the initial outbreak (eg, if interviewers became better skilled in eliciting risk over time) could have biased our estimates. Third, unlike our previous analysis, there was no information on homelessness status, so we were unable to include this population in our model; however, experiencing homelessness has been observed as an independent risk in other settings, such as San Diego, California [35]. Fourth, we modeled at the state level and assumed homogeneous mixing, which is a simplifying assumption. Although we confirmed that at the state level the epidemics followed singular trajectories, we acknowledge that these epidemics were likely driven by county-level (or even smaller) subepidemics. Fifth, we used data on reported HAV cases that only represent a fraction of true cases. In our analysis, we assumed that case acquisition of HAV infection during outbreaks was consistent over time and that reporting did not have systematic biases over time or population. Sixth, we assumed the national PWID population size estimate was distributed by state similar to the distribution of PWUD by state. Although in our previous analysis in Louisville, we found that the herd immunity threshold was not particularly sensitive to this parameter [19], a more robust population size estimate would improve our estimates. Seventh, we noted that although the I^2^ was large (proportion of the variance in the observed effect due to variance in true effects), meta-analyses of prevalence estimates commonly yield high I^2^ (an analysis of 134 meta-analyses of prevalence studies found a median I^2^ of 97% [28]). Guidance from the Cochrane group is to focus on the prediction interval to account for this heterogeneity, the upper bound of which we used to inform our recommended threshold estimate [28–30]. Finally, we determined a critical threshold target based on vaccination alone. If accurately accounted for, past infection could be counted and thus reduce the critical vaccination coverage given the generated immunity. However, in practice, public health programs are unlikely to be able to test individuals or search medical records for evidence of past infection prior to immunization. Therefore, for practical purposes, the vaccination threshold presented would need to be achieved in order to ensure herd immunity is reached, although this may be a conservative approach given some baseline immunity.

In conclusion, we find that hepatitis A vaccination programs in the United States will need to achieve vaccination coverage of at least 80% among PWID to prevent most HAV outbreaks among this population. Further efforts to increase vaccination coverage among PWID and PWUD more broadly are needed to reduce cases and improve health equity in this group.

## Supplementary Data

Supplementary materials are available at *Clinical Infectious Diseases* online. Consisting of data provided by the authors to benefit the reader, the posted materials are not copyedited and are the sole responsibility of the authors, so questions or comments should be addressed to the corresponding author.

## Supplementary Material

ciad552_Supplementary_Data

## Notes

***Acknowledgments*.** The authors thank the local and state health department staff who investigated hepatitis A cases and contributed data to the National Notifiable Diseases Surveillance System for this analysis. The authors also thank Megan Hofmeister for her manuscripts edits, and Julian Higgins for his helpful advice on interpretation of the meta-analysis.

***Financial support*.** This work was supported by the Centers for Disease Control and Prevention, National Center for HIV/AIDS, Viral Hepatitis, STD, and TB Prevention (Epidemiologic and Economic Modeling agreement NU38PS004650). N. C. L. is supported by the NIH National Institute of Allergy and Infectious Diseases (NIAID) New Innovator Award (DP2AI170485). C. A. D. is supported by a UK National Institute for Health Research (NIHR) grant paid to their institution (Health Protection Research Unit in Emerging and Zoonotic Infections, NIHR200907).
