# Supplementary material for: Determining Herd Immunity Thresholds for Hepatitis A Virus Transmission to Inform Vaccination Strategies Among People Who Inject Drugs in 16 US States
Source: Clin Infect Dis. 2023 Sep 21;78(4):976–82. doi: 10.1093/cid/ciad552 (PMC11006109; doi:10.1093/cid/ciad552)
Supplement: ciad552_Supplementary_Data [file ciad552_supplementary_data.docx]

**SUPPLEMENTARY INFORMATION**

We used a previously published deterministic compartmental model(1), which classifies individuals in the population according to their infection status. At any time, *t*, an individual may be in one of the following states: susceptible ($S$), latent ($L$), infectious ($I$), temporary remission ($R$) or immune ($Z$). Individuals in the $S$ state are susceptible to the disease, individuals in the *L* state have the disease but are not yet infectious, individuals in the $I$state are infectious, individuals in the $Z$ state are immune to the disease as a result of vaccination or past infection and individuals in the $R$ state will experience a relapse of symptoms at some point in the future.

The model equations are:

|  | $\frac{dS}{dt}=-\frac{\beta SI}{N}$  $\frac{dL}{dt}= \frac{\beta SI}{N}-\alpha L$  $\frac{dI}{dt}=\alpha L+\sigma R-\gamma I$  $\frac{dZ}{dt}=\eta\gamma I$  $\frac{dR}{dt}=\left( 1-\eta\right)\gamma I-\sigma R$ | (S1) |
| --- | --- | --- |

where the parameters 1/α, 1/γ, 1/σ, 1 −η, $\beta$ represent duration of latency, duration of infectiousness, duration of the remission phase, proportion of relapsing individuals, and effective contact rate, respectively.

We computed the number of detected HAV cases at day $t$, $y(t)$, as

$y\left( t \right)=\int_{t}^{t+1} \kappa(\alpha L(s)+\sigma R(s)) ds$, (S2)

where $\kappa$ is the rate of case detection.

As discussed in(1). the model as expressed in equations (S1) and (S2) is unidentifiable. To ensure identifiability of the model, we reparametrized the model equations.

Consider $S_{1}=S\times\kappa$, $L_{1}=L\times\kappa,$ $I_{1}=I\times\kappa,$ $R_{1}=R\times\kappa$, $Z_{1}=Z\times\kappa,$and $\beta_{1} =\beta/\kappa$. Substituting these expressions into equations (S1) and (S2), we have

| $\frac{dS_{1}}{dt}=-\frac{\beta_{1}S_{1}I_{1}}{N}$ $\frac{dL_{1}}{dt}= \frac{\beta_{1}S_{1}I_{1}}{N}-\alpha L_{1}$  $\frac{dI_{1}}{dt}=\alpha L_{1}+\sigma R_{1}-\gamma I_{1}$  $\frac{dZ_{1}}{dt}=\eta\gamma I_{1}$  $\frac{dR_{1}}{dt}=\left( 1-\eta\right)\gamma I_{1}-\sigma R_{1}$ | (S3) |
| --- | --- |

and

$y_{1}\left( t \right)=\int_{t}^{t+1} \alpha L_{1}(s)+\sigma R_{1}(s)) ds$. (S4)

With this reparameterization, the model now represents the dynamics for observed cases, rather than true cases, and all parameters are locally structurally identifiable.

**Parameter estimation**

*Likelihood function*

Assuming a Poisson likelihood for the observed case counts $\boldsymbol{x}=\{x_{1},\ldots x_{k}\}$ (outbreak among PWID until the peak at *k* weeks), we estimated the parameters of the reparametrized model (Table 1, main text) via maximum likelihood estimation (MLE). Let $\boldsymbol{\theta}$ represent the set of parameters to be estimated. The likelihood function given $\boldsymbol{x}$ is

$L\left( \boldsymbol{\theta} \right)= \prod_{t=1}^{t=k} \frac{y_{t}^{x_{t}}e^{-y_{t}}}{x_{t}!}$,

where $y_{t}>0$ is the model outcome at day $t$.

MLE enables the estimation of the set of parameter values $\hat{\boldsymbol{\theta}}$ which maximizes $L(\boldsymbol{\theta})$ and thus best describes the observed epidemic. Uncertainty quantification for the estimated parameters is described in the main text.

*Initial conditions*

Following [2], we chose the initial condition for $L_{1}$, $L_{1}(0)$, to be in equilibrium with $I_{1}(0)$. The initial conditions for all states are as follows$: I_{1}(0)= I_{0}, L_{1}(0)=I_{0}\alpha/\gamma,$ $Z_{1}\left( 0 \right)=\epsilon N$, $R_{1}\left( 0 \right)=0$ and $S_{1}\left( 0 \right)=N-L_{1}\left( 0 \right)-I_{1}\left( 0 \right)-Z_{1}\left( 0 \right)-R_{1}\left( 0 \right).$

**Supplementary Table 1**: Descriptions, values and references for model parameters.

#

| **Description** | **Symbol** | **Value** | **Reference** |
| --- | --- | --- | --- |
| Effective contact rate at the start of the outbreak | $\beta$ |  | Estimated through model calibration |
| Number of infectious individuals in week 0 | $I_{1}(0)$ |  | Estimated through model calibration |
| Duration of latent period (in weeks) | 1/α | 1.57 | (2) |
| Duration of infectious period (in weeks) | 1/γ | 4.64 | (2) |
| Duration of remission period (in weeks) | 1/σ | 4.30 | (3, 4) |
| Probability of experiencing a relapse | 1 −η | 0.11 | (3, 4) |
| Proportion of initially immune individuals | $\varepsilon$ | 0.43 | (5, 6) |
| PWID population size | *N* | Varied by State, see supplementary table 3 (Excel) | National estimate (3.69 million) allocated by state, see text(7, 8) |

**Supplementary Table 2**: **PWID population size estimates by state used for the model.** Bolded states are the states used in the modeling.

| **State** | **Population age 18+ July 1 2018** | **% drug use age 18+ from 2018 SAMHSA** | **# PWUD** | **% of U.S. PWUD** | **# of PWID (point estimate)** | **# PWID, lower bound** | **# PWID, upper bound** |
| --- | --- | --- | --- | --- | --- | --- | --- |
| **.Alabama** | **3,798,031** | **11.04%** | **419185** | **0.014** | **51833** | **26274** | **102044** |
| .Alaska | 553,622 | 19.02% | 105311 | 0.004 | 13022 | 6601 | 25636 |
| .Arizona | 5,528,989 | 12.98% | 717892 | 0.024 | 88770 | 44996 | 174759 |
| **.Arkansas** | **2,310,645** | **11.11%** | **256634** | **0.009** | **31734** | **16085** | **62473** |
| .California | 30,567,090 | 13.94% | 4260285 | 0.143 | 526798 | 267028 | 1037098 |
| .Colorado | 4,430,329 | 20.51% | 908855 | 0.030 | 112383 | 56966 | 221246 |
| .Connecticut | 2,837,472 | 14.71% | 417531 | 0.014 | 51629 | 26170 | 101641 |
| .Delaware | 763,555 | 13.43% | 102555 | 0.003 | 12681 | 6428 | 24965 |
| .District of Columbia | 574,961 | 20.44% | 117547 | 0.004 | 14535 | 7368 | 28615 |
| **.Florida** | **17,070,244** | **10.91%** | **1862606** | **0.062** | **230317** | **116745** | **453421** |
| .Georgia | 8,013,724 | 9.80% | 785179 | 0.026 | 97090 | 49214 | 191139 |
| .Hawaii | 1,117,077 | 10.60% | 118409 | 0.004 | 14642 | 7422 | 28825 |
| .Idaho | 1,307,236 | 9.68% | 126505 | 0.004 | 15643 | 7929 | 30796 |
| .Illinois | 9,883,814 | 11.29% | 1116195 | 0.037 | 138021 | 69961 | 271720 |
| .Indiana | 5,123,748 | 12.28% | 629244 | 0.021 | 77808 | 39440 | 153179 |
| .Iowa | 2,425,378 | 9.60% | 232953 | 0.008 | 28805 | 14601 | 56709 |
| .Kansas | 2,205,544 | 9.07% | 199987 | 0.007 | 24729 | 12535 | 48684 |
| **.Kentucky** | **3,459,573** | **10.53%** | **364347** | **0.012** | **45053** | **22837** | **88695** |
| **.Louisiana** | **3,564,062** | **9.96%** | **354809** | **0.012** | **43873** | **22239** | **86373** |
| .Maine | 1,088,000 | 18.05% | 196330 | 0.007 | 24277 | 12306 | 47793 |
| .Maryland | 4,702,570 | 11.82% | 555905 | 0.019 | 68739 | 34843 | 135326 |
| **.Massachusetts** | **5,535,291** | **16.25%** | **899300** | **0.030** | **111201** | **56367** | **218920** |
| **.Michigan** | **7,831,247** | **14.87%** | **1164490** | **0.039** | **143993** | **72988** | **283476** |
| .Minnesota | 4,308,564 | 10.96% | 472080 | 0.016 | 58374 | 29589 | 114920 |
| **.Mississippi** | **2,280,389** | **8.96%** | **204243** | **0.007** | **25255** | **12802** | **49720** |
| .Missouri | 4,749,622 | 10.49% | 498019 | 0.017 | 61582 | 31215 | 121235 |
| .Montana | 832,871 | 16.25% | 135336 | 0.005 | 16735 | 8483 | 32945 |
| .Nebraska | 1,452,427 | 9.74% | 141499 | 0.005 | 17497 | 8869 | 34446 |
| .Nevada | 2,345,395 | 16.96% | 397834 | 0.013 | 49193 | 24936 | 96846 |
| .New Hampshire | 1,098,288 | 15.89% | 174525 | 0.006 | 21581 | 10939 | 42485 |
| .New Jersey | 6,954,877 | 9.41% | 654558 | 0.022 | 80938 | 41027 | 159342 |
| **.New Mexico** | **1,613,275** | **14.47%** | **233472** | **0.008** | **28870** | **14634** | **56835** |
| **.New York** | **15,474,107** | **11.68%** | **1807144** | **0.060** | **223459** | **113269** | **439920** |
| **.North Carolina** | **8,082,975** | **9.37%** | **757487** | **0.025** | **93666** | **47478** | **184398** |
| .North Dakota | 581,379 | 8.98% | 52188 | 0.002 | 6453 | 3271 | 12704 |
| **.Ohio** | **9,096,117** | **10.28%** | **935492** | **0.031** | **115677** | **58635** | **227731** |
| .Oklahoma | 2,986,593 | 9.71% | 289962 | 0.010 | 35855 | 18174 | 70587 |
| .Oregon | 3,317,146 | 20.80% | 690039 | 0.023 | 85326 | 43251 | 167979 |
| .Pennsylvania | 10,158,149 | 10.14% | 1030252 | 0.034 | 127394 | 64575 | 250798 |
| .Rhode Island | 852,102 | 17.24% | 146943 | 0.005 | 18170 | 9210 | 35771 |
| **.South Carolina** | **3,978,182** | **10.04%** | **399438** | **0.013** | **49392** | **25036** | **97237** |
| .South Dakota | 664,629 | 8.62% | 57274 | 0.002 | 7082 | 3590 | 13942 |
| **.Tennessee** | **5,263,790** | **10.24%** | **538805** | **0.018** | **66625** | **33771** | **131163** |
| .Texas | 21,303,746 | 8.06% | 1716107 | 0.057 | 212202 | 107563 | 417759 |
| **.Utah** | **2,228,643** | **7.80%** | **173856** | **0.006** | **21498** | **10897** | **42322** |
| .Vermont | 510,326 | 20.82% | 106272 | 0.004 | 13141 | 6661 | 25870 |
| **.Virginia** | **6,647,893** | **8.80%** | **584761** | **0.020** | **72308** | **36652** | **142351** |
| .Washington | 5,872,306 | 18.51% | 1087190 | 0.036 | 134435 | 68143 | 264659 |
| **.West Virginia** | **1,441,672** | **10.86%** | **156611** | **0.005** | **19365** | **9816** | **38124** |
| .Wisconsin | 4,537,465 | 10.67% | 484361 | 0.016 | 59893 | 30359 | 117910 |
| .Wyoming | 442,962 | 9.06% | 40125 | 0.001 | 4962 | 2515 | 9768 |

**Supplementary Table 3. Sensitivity analysis performing the meta-analysis on the R_0_ for estimation of herd immunity threshold.**

| State | R_0_ | [95% conf. | interval] | % Weight |
| --- | --- | --- | --- | --- |
|  |  |  |  |  |
| Alabama | 2.23 | 2.06 | 2.41 | 6.94 |
| Arkansas | 2.78 | 2.15 | 3.42 | 5.64 |
| Florida | 2.57 | 2.48 | 2.65 | 7.05 |
| Indiana | 3.06 | 2.71 | 3.42 | 6.56 |
| Kentucky | 2.42 | 2.35 | 2.49 | 7.06 |
| Louisiana | 2.81 | 2.62 | 3.01 | 6.91 |
| Massachusetts | 3.49 | 3.00 | 3.98 | 6.14 |
| Mississippi | 2.64 | 2.29 | 2.98 | 6.59 |
| New Mexico | 2.40 | 1.78 | 3.03 | 5.67 |
| New York | 4.57 | 2.80 | 6.34 | 2.37 |
| North Carolina | 2.20 | 1.88 | 2.52 | 6.65 |
| Ohio | 2.98 | 2.80 | 3.17 | 6.93 |
| Tennessee | 2.38 | 2.31 | 2.45 | 7.05 |
| Utah | 2.99 | 2.51 | 3.48 | 6.16 |
| Virginia | 2.56 | 2.14 | 2.97 | 6.38 |
| West Virginia | 5.02 | 4.45 | 5.58 | 5.90 |
|  |  |  |  |  |
| Pooled | 2.86 | 2.52 | 3.19 | 100 |

**Supplementary Figure 1. Calibrated model fits for 16 U.S. states

.** Black dots show the observed HAV new diagnoses among PWID. Blue line shows the maximum likelihood model fits, with shading representing the 95% CI model estimate. Note the y axis varies. HAV: hepatitis a virus; PWID: people who inject drugs.
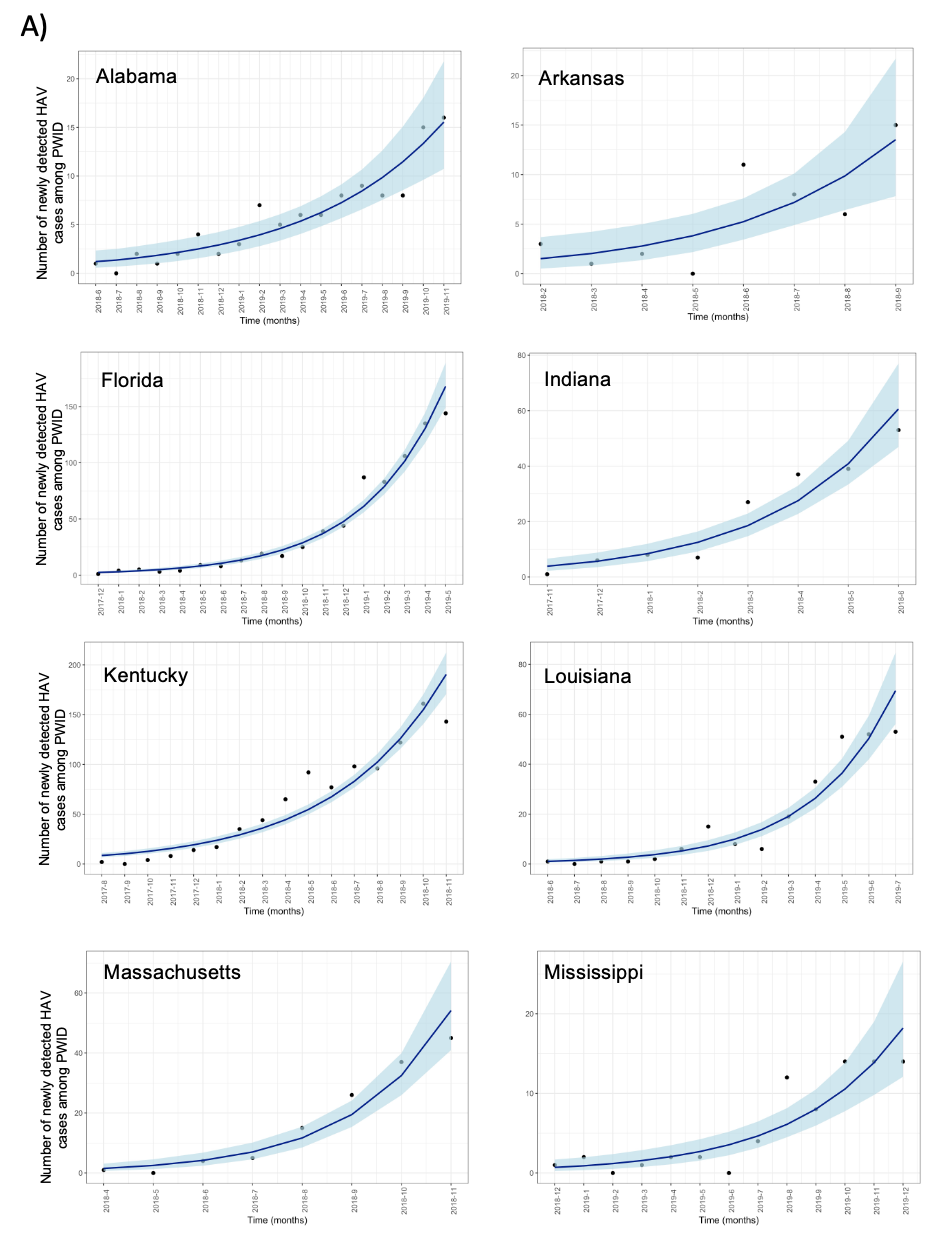


**
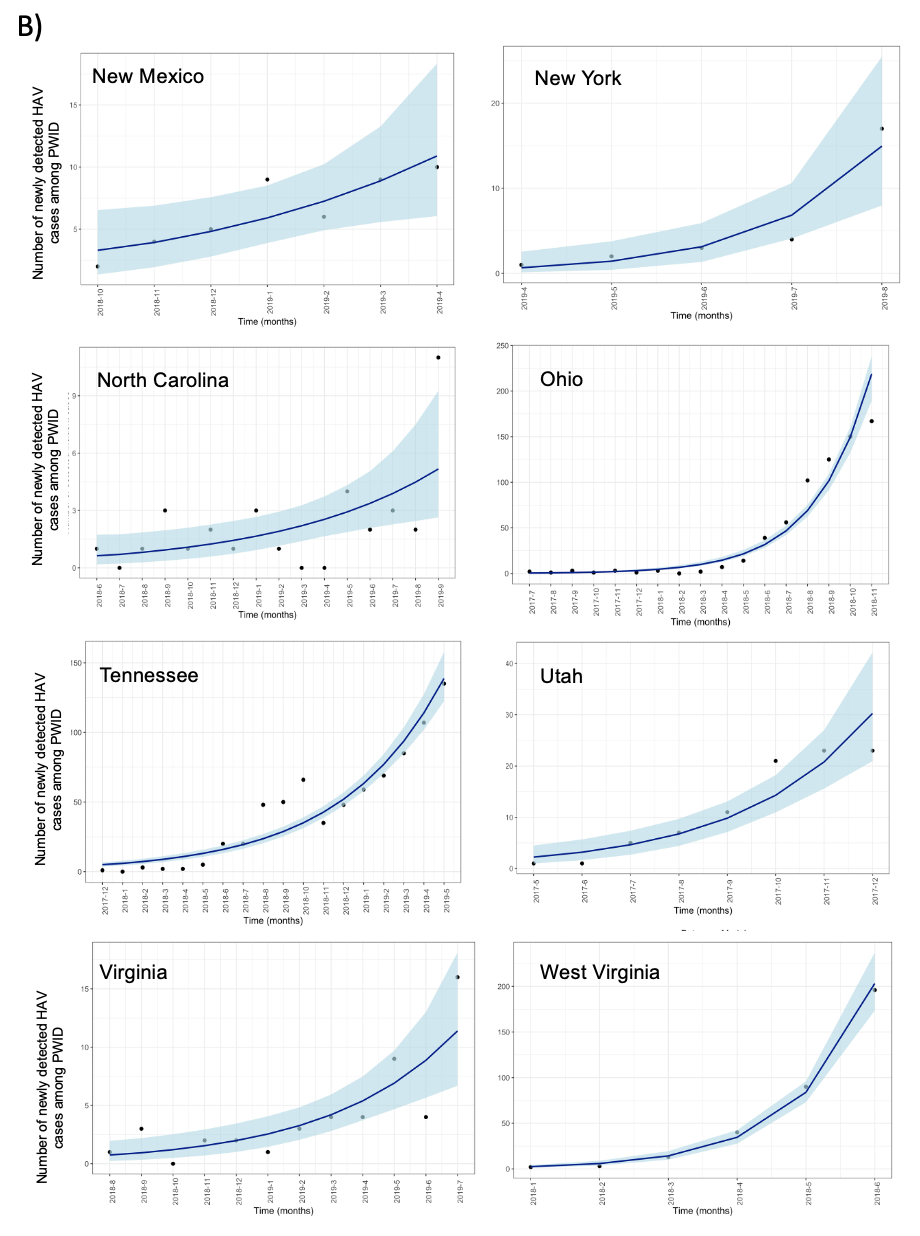
**

**Supplementary Figure 2. Sensitivity analysis on median herd immunity thresholds in each of the 16 U.S. states.** Baseline analysis (black bars) excludes cases with missing drug use data, sensitivity analysis (grey bars) include cases with missing drug use data (see methods)**.** HAV: hepatitis A virus; PWID: people who inject drugs.

**
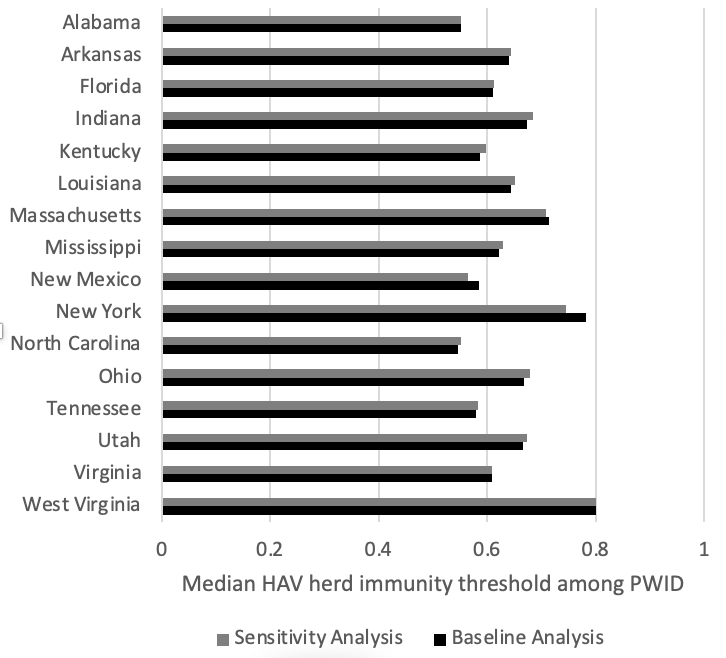
**

1. Dankwa EA, Donnelly CA, Brouwer AF, Zhao R, Montgomery MP, Weng MK, et al. Estimating vaccination threshold and impact in the 2017-2019 hepatitis A virus outbreak among persons experiencing homelessness or who use drugs in Louisville, Kentucky, United States. Vaccine. 2021;39(49):7182-90.

2. Centers for Disease Control and Prevention. Epidemiology and prevention of vaccine-preventable diseases. 14th ed. Hall EW, A.P.; Hamborsky, J; et al., editor. Washington, D.C.: Public Health Foundation; 2021.

3. Schiff ER. Atypical clinical manifestations of hepatitis A. Vaccine. 1992;10 Suppl 1:S18-20.

4. Glikson M, Galun E, Oren R, Tur-Kaspa R, Shouval D. Relapsing hepatitis A. Review of 14 cases and literature survey. Medicine (Baltimore). 1992;71(1):14-23.

5. Figgatt M, Hildick-Smith J, Addish E, Coleman J, Benitez J, Freeland C, et al. Susceptibility to Hepatitis A and B Virus Among Clients at a Syringe Services Program in Philadelphia, 2018. Public Health Rep. 2020;135(5):691-9.

6. Koepke R, Sill DN, Akhtar WZ, Mitchell KP, Guilfoyle SM, Westergaard RP, et al. Hepatitis A and Hepatitis B Vaccination Coverage Among Persons Who Inject Drugs and Have Evidence of Hepatitis C Infection. Public Health Rep. 2019;134(6):651-9.

7. Bradley H, Hall EW, Asher A, Furukawa NW, Jones CM, Shealey J, et al. Estimated Number of People Who Inject Drugs in the United States. Clin Infect Dis. 2023;76(1):96-102.

8. SAMHSA. 2017-2018 National Survey on Drug Use and Health: Model-Based Prevalence Estimates (50 States and the District of Columbia). 2018.
